# Comparison of Vitamin D Levels in Premature Infants with and without Retinopathy of Prematurity

**DOI:** 10.34172/aim.2022.36

**Published:** 2022-04-01

**Authors:** Hassan Boskabadi, Majid Abrishami, Nasser shoeibi, Zahra sanei, Ali Moradi, Maryam Zakerihamidi

**Affiliations:** ^1^Department of Pediatrics, Faculty of Medicine, Mashhad University of Medical Sciences, Mashhad, IR Iran; ^2^Eye Research Center, School of Medicine, Mashhad University of Medical Sciences, Mashhad, IR Iran; ^3^School of Medicine, Mashhad University of Medical Sciences, Mashhad, IR Iran; ^4^Orthopedic Research Center, Ghaem Hospital, Mashhad University of Medical Sciences, Mashhad, Iran; ^5^Clinical Research Development Unit, Ghaem Hospital, Mashhad University of Medical Sciences, Mashhad, Iran; ^6^Department of Midwifery, School of Medical Sciences, Islamic Azad University, Tonekabon, Iran

**Keywords:** Birth weight, Gestational age, Retinopathy of prematurity, Vitamin D

## Abstract

**Background:**

Retinopathy of prematurity (RoP) is a cause of newborn blindness. Several predisposing factors have been reported to contribute to the disease process. The current study aimed to compare serum vitamin D levels in infants with and without RoP.

**Methods:**

This case-control study was conducted on 154 very low birth weight (VLBW) infants admitted to Ghaem hospital, Mashhad, Iran, during 2016–2019. Retinal examination for RoP was done at the 32nd week of pregnancy and vitamin D level was determined using the infants’ first-day serum samples. A researcher-made questionnaire including maternal, infant, laboratory, and retinal examination information was used as the data collection tool.

**Results:**

Out of 154 infants in the study, 56 (36.4%) were normal while 98 (63.6%) had RoP. Based on the severity of retinopathy, 43 infants (43.9%) were at stage I, 48 (49%) at stage II, and 7 (7.1%) at stage III. Significant differences in neonatal (*P*<0.001) and maternal (*P*=0.015) vitamin D levels, first and fifth minute Apgar scores (*P*=0.034 and *P*=0.001, respectively), and weight (*P*=0.014) were found between the infants with and without RoP.

**Conclusion:**

The incidence of RoP was higher in infants with lower gestational age, lower birth weight, low first and fifth minutes Apgar scores, and male sex. Low serum levels of vitamin D in premature infants and their mothers were associated with incidence of RoP. The higher the stage of RoP, the greater was the severity of vitamin D deficiency. Thus, controlling the maternal vitamin D level during pregnancy, consumption of vitamin D supplements, and investigation of serum vitamin D levels in premature infants are recommended. Early correction of vitamin D deficiency may lead to reduction of RoP.

## Introduction

 With the advancing knowledge of perinatologyin recent decades, maintaining the survival of high-risk neonates including premature infants has been made possible.^1^ Very low birth weight (VLBW) infants have many more problems and require greater care.^2^ Retinopathy of Prematurity (RoP).^3^ is one of the serious problems among VLBW infants and a leading cause of childhood blindness. RoP, as a side effect of oxygen therapy, can lead to blindness in the incompletely vascularized retina in premature infants.^4^

 The incidence and severity of RoP increases by reduction in gestational age and birth weight. In a previous study in Iran, the prevalence of RoP in infants under 32 weeks was reported at about 44%.^2^ Although many factors are involved in the incidence of RoP, low gestational age, LBW, and oxygen use are important factors in developing RoP. Other possible important factors include: apnea, mechanical ventilation, anemia and need for blood transfusion, vitamin deficiencies, intraventricularhemorrhage, sepsis, acidosis, hypotension, pneumothorax, chronic lung diseases, high levels of arterial carbon dioxide, seizure, and bradycardia.^5,6^ Effective early identification of high risk infants and examination of their retina has been proven to reduce the complications of RoP.^7,8^

 Maintaining normal blood levels of calcium and phosphorus is the main biological function of vitamin D. Vitamin D promotes an overall boost in the immune system. Considering the numerous biologic functions of vitamin D in body metabolism, the need for it increases during pregnancy. Moreover, the fetus is unable to produce 25-hydroxy vitamin D and relies completely on its maternal transfer.^9^ Also, as placental transfer of vitamin D mostly occurs during the third trimester, vitamin D deficiency is one of the problems of premature infants.^10^ Maternal vitamin D deficiency leads to reduction in placental vitamin D transfer.^11^

 The risk factors for maternal and consequently neonatal vitamin D deficiency include low exposure to sunlight, regular sunscreen use, residence in northern latitudes, dark skin, obesity, multilayer clothing, aging, malnutrition, malabsorption syndrome, and medications.^12^ In neonates, vitamin D deficiency accompanies intrauterine growth restriction, weight loss, and increased risk of preterm labor, leading to preterm labor, respiratory distress syndrome, and increased bronchopulmonary dysplasia.^13^ Low serum levels of 25-hydroxy vitamin D on the first day of life have been proposed as a potential risk factor for retinopathy of immaturity, and hence, treatment of immature neonates is necessary.^14^ Expression of vitamin D receptor has been shown to play an important role in the evolution of retinal vessels, especially during the final steps of retinal vascularization. Although pathologic retinal neovascularization during oxygen-therapy induced ischemic retinopathy is independent of the expression of vitamin D receptor, the inhibition of retinal neovascularization through 1,25 hydroxy vitamin D3 depends on the expression of vitamin D receptor.^15^

 Considering the placental passage of the highest levels of calcium and vitamin D during the third trimester, premature infants are most likely to have problems in this regard. On the other hand, these problems would be exacerbated in cases of maternal vitamin D deficiency. As vitamin D deficiency can be a potential risk factor for RoP, the current case-control study attempted to evaluate the vitamin D levels in infants with and without RoP.

## Materials and Methods

 This study was conducted on VLBW infants admitted to the intensive care unit at Ghaem hospital, Mashhad, Iran, during 2016–2019.

 Due to the lack of a similar study and according to Kabataş et al^14^ who reported the vitamin D level in infants with RoP who needed or did not need treatment at 7.1 ± 5.2 ng/dL and 11.9 ± 6.5 ng/dL, respectively, using the student *t *test with α = 0.01 and β = 0.1, the sample size in each group was calculated as 46 infants. However, considering the probable dropout, the final sample size was adjusted to 56 infants in each group.

 Prepartum maternal whole blood samples (2 mL) were collected. Serum samples were also taken from infants < 1500 g for vitamin D measurement along with sampling for other routine tests in the first hour of life. The serum samples were stored at -20°C for further laboratory evaluation. Vitamin D measurement was performed by ELISA (RT2100C, Germany). The VLBW infants were allocated into four groups according to their vitamin D levels: severe deficiency (≤ 10 ng/mL), moderate deficiency (10.1–20 ng/mL), mild deficiency (20.1–30 ng/mL) and normal (> 30 ng/mL).

 The demographic data of infants were recorded in a checklist. The characteristics of infants (birth weight, age, gender, gestational age, Apgar score, and clinical symptoms), maternal history (age, pregnancy and delivery problems, mode of delivery), and laboratory results were collected and recorded in a questionnaire. The infant’s condition was monitored up to discharge time. Screening for RoP was started between 4–6 weeks after birth. A single drop of cyclopentolate hydrochloride/phenylephrine (2 mg.mL ^-1^/2.5 mg.mL ^-1^) was distilled into each eye 30 minutes before the examination to induce mydriasis. Fundal examination using an indirect ophthalmoscope and a 20D lens was performed by a single investigator (MA) at Khatam-al-Anbia hospital, a tertiary referral eye hospital. The examinations continued until full vascularization of the retina or the post-conception age of 40 weeks according to an accurate gestational age. Cases with RoP were treated according to the severity and diagnostic criteria. The infant was allocated in the control group if the primary retinal examination was normal. RoP was defined based on the international classification.^16^

 The severity of RoP was defined in five stages through which the disease serially passes. However, stages I and IV may be present simultaneously in one eye^17^:

- Stage I (presence of a demarcation line)
- Stage II (presence of ridge ± fibrous - vesicular proliferative small tissues)
- Stage III (ridge with extra-retinal fibro-vesicular proliferation)
- Stage IV (retinal sub-total detachment A, without fovea involvement & B with foveal involvement)
- Stage V (total retinal detachment).

Plus disease is the severity index of the disease and is defined as intravascular dilation and arteriolar tortuosity of the posterior vessels.^18^

 Following data collection and recording in SPSS 21, normality (Kolmogorov-Smirnov) test, Pearson’s correlation coefficient, and independent t test were used for analysis of the relationship between the variables. The Kolmogorov-Smirnov test was used to assess the normality of data distribution. In case of non-normal distribution, non-parametric tests including Spearman’s correlation coefficient and Mann-Whitney tests were used.

 The association of the delivery and gender with RoP condition was assessed using the chi-square or Fisher’s exact tests. After checking the homogeneity of variances (Levene’s test), analysis of co-variances was used to control the gestational age, first and fifth minute Apgar scores, and duration of O_2_ therapy.* P* < 0.05 was considered as the minimum significant level in this study.

## Results

 Among 154 participants in the study, 56 infants (36.4%) were normal and 98 infants (63.6%) had RoP. Based on the severity of retinopathy, 43 infants (43.9%) were stage I, 48 infants (49%) were stage II, and 7 infants (7.1%) were stage III. Plus disease was present in five cases (5.1%). In terms of zone of RoP, 3.8% was zone 1, 46.3% zone 2, 45% zone 3, 1.3% zone 1–2, and 3.8% was zone 2–3. In the follow-up of infants with RoP, 91 infants improved, five infants needed bevacizumab (Avastin) injection, and two patients had surgery. In terms of RoP prognosis, 96.5% were normal and 3.5% progressed to blindness.

 Statistically significant differences were found between the infants with and without RoP in terms of neonatal vitamin D (*P* < 0.001, CI: 7.96–13.79), maternal vitamin D (*P* = 0.015), gestational age (*P* < 0.001, CI: 2.12–18.73), first minute Apgar score (*P* = 0.034, CI: 0.05–1.37), fifth minute Apgar score (*P* < 0.001, CI: 0.29–1.31), and birth weight (*P* = 0.014, CI: 35.75–279.0) (Table 1). Analysis of co-variances with the elimination of factors affecting the gestational age, first and fifth minute Apgar scores, and duration of O_2_ therapy showed a significant difference in the serum vitamin D level (*P* = 0.013) between the RoP and normal neonates.

**Table 1:** Comparison of Average Neonatal and Maternal Variables between Groups of Infants with and without RoP

**Variables**	**Infants without RoP** **56 (36.4%)**	**Infants with RoP** **98 (63.6%)**	**Mean Differences (95% CI)**	***P *** **Value** ^a^
Neonatal vitamin D (ng/dL)	22.33 ± 11.40	11.46 ± 6.50	10.87 (7.96–13.79)	0.000
Maternal vitamin D (ng/dL)	29.66 ± 14.49	19.23 ± 14.47	10.43 (2.12–18.73)	0.015
Gestational age (wk)	32.05 ± 1.90	30.58 ± 2.20	1.47 (0.76–2.17)	0.000
First minute Apgar score	6.87 ± 1.81	6.15 ± 2.12	0.72 (0.05–1.37)	0.034
Fifth minute Apgar score	8.36 ± 1.11	7.55 ± 1.66	0.81 (0.29–1.31)	0.001
Birth weight	1491.87 ± 382.80	1334.58 ± 353.85	157.29 (35.75–279.0)	0.014

RoP, Retinopathy of prematurity. Values are in terms of mean ± SD; ^a^*t* test.

 About 59% of infants with RoP and 63% of those without RoP were born through cesarean section. In addition, 51% of infants with RoP and 43% of those without RoP were male. Although cesarean delivery (*P* = 0.769) and male gender (*P* = 0.352) were more prevalent among infants with RoP, the difference between the two groups was not statistically significant. According to Table 2, the higher the RoP grade, the lower was the vitamin D level.

**Table 2:** Average Vitamin D Levels at Different **Retinopathy of Prematurity** Severities

**Global Exam**	**Mean±SD**
Normal	22.33 ± 11.40
RoP 1	14.06 ± 7.79
RoP 2	11.43 ± 7.49
RoP 3	8.20 ± 0.14

ROP, retinopathy of prematurity.

**Figure 1:**
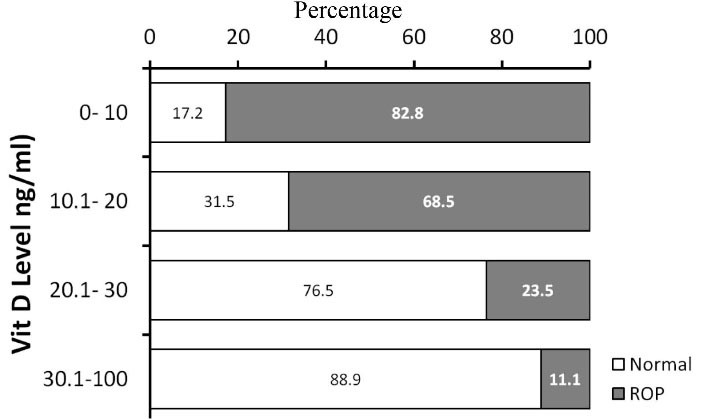
Distribution of RoP among Infants with Different Levels of Vitamin D.

 Vitamin D deficiency showed a significant association with the incidence of RoP (Pearson’s correlation = -0.518, *P* < 0.001). Almost 83% of infants with severe Vit D deficiency suffered from RoP while 17.2% had normal retina; however, only 11.1% of infants with normal vitamin D levels had RoP and 88.9% had normal retina (Figure 1). The prevalence of strabismus increased by 20% in LBW infants, and this increase was related to the RoP stage, as the incidence of strabismus was 6% in stage I and 30% in stage III RoP.

## Discussion

 This study aimed to assess the relationship between neonatal serum vitamin D levels and RoP. According to our findings, infants with RoP had significantly lower serum vitamin D levels compared to those without RoP. Also, vitamin D level was strongly associated with the incidence of RoP (Pearson correlation: 0.518). The severity of RoP increased with reduction in serum vitamin D. Therefore, vitamin D deficiency can be suggested as a risk factor for prediction of neonatal RoP.

 A significant relationship was shown between the incidence of RoP and infant’s weight. The prevalence of RoP was higher with lower birth weight. This result is consistent with the findings of several previous studies.^19-22^

 Forty-four percent of our newborns had RoP stage I, 49% stage II, and 7.1% stage III. The prevalence of RoP in a previous study was 68% in stage I, 23.5% in stage II, and 8.5% in stage III.^23^ Also, another study reported that 30.2% of infants under 1500 g suffered from stage I and II RoP and 16.6% of them suffered from stage III and above. In infants weighing 1500 to 2500 g, 3.2% had stage I and II RoP, and 0.6% had stage III and above.^24^

 Consistent with the findings of a previous report, the incidence of RoP in our study was higher at lower gestational ages.^19^ Low gestational age, LBW, and receiving oxygen therapy are risk factors for the incidence of RoP.^25^ The incidence of RoP has been reported at 44% in infants with gestational age lower than 30–32 weeks or a birth-weight less than 1500 g.^26,27^

 In the follow-up phase, 92.86% of infants with RoP improved, 5.1% needed Avastin injections, and 2.04% had surgery. Fortunately, RoP improved without visual impairment in most cases (80%)^3^ The prevalence of strabismus increased in LBW infants by 20%. This increase was related with the RoP stage, as the incidence of strabismus in stage I RoP was 6%, while, it increased to 30% in stage III. However, early treatment has been shown to improve the vision even in the eyes with poor visual function.^28^ In a previous study with 70% RoP in premature infants, 68% recovered and 32% needed treatment for RoP (75% of infants were treated by Avastin injection and 25% by laser).^29^

 Consistent with other studies, our findings showed no significant relationship between gender and RoP incidence.^30,31^

 Serum levels of vitamin D in infants with RoP were lower in our study regardless of gestational age. The severity of vitamin D deficiency increased with higher stages of RoP. To the best of our knowledge, no relationship between vitamin D deficiency and RoP has been reported before. As vitamin D directly affects the vascular endothelial stability, its deficiency will most likely affect the incidence of RoP in premature infants who are at risk of vitamin D deficiency.^32^ The inflammatory and angiogenic effects of vitamin D deficiency can cause early damage to the retinal blood vessels.^33^ *In vitro* studies have shown that vitamin D improves the angiogenic properties of precursor cells through upregulation of the expression of vascular endothelial growth factor. Since vitamin D deficiency is associated with degeneration of endothelial function, the number of endothelial progenitor cells can also be reduced.^34^ The possible mechanisms by which vitamin D can cause small vessels damage include inflammation, fat metabolism, and the renin-angiotensin-aldosterone system.^35^

 As a limitation, the present study failed to consider the gestational age and birth weight of the participant neonates. Future studies with subject matching in terms of gestational age and birth weight of neonates are recommended.

 In conclusion, the incidence of RoP was higher in infants with lower gestational age, lower birth weight, low first and fifth minutes Apgar scores, and male sex. Low serum levels of vitamin D in premature infants and their mothers were associated with incidence of RoP. The higher the stage of RoP, the greater was the severity of vitamin D deficiency. Thus, controlling the maternal vitamin D level during pregnancy, consumption of vitamin D supplements, and investigation of serum vitamin D levels in premature infants are recommended. Early correction of vitamin D deficiency may lead to reduction of RoP.
